# Proteasome Inhibitors Alter Levels of Intracellular Peptides in HEK293T and SH-SY5Y Cells

**DOI:** 10.1371/journal.pone.0103604

**Published:** 2014-07-31

**Authors:** Sayani Dasgupta, Leandro M. Castro, Russell Dulman, Ciyu Yang, Marion Schmidt, Emer S. Ferro, Lloyd D. Fricker

**Affiliations:** 1 Department of Molecular Pharmacology, Albert Einstein College of Medicine, Bronx, New York, United States of America; 2 Department of Pharmacology, Support Center for Research in Proteolysis and Cell Signaling, Biomedical Sciences Institute, University of São Paulo, São Paulo, SP, Brazil; 3 Department of Biochemistry, Albert Einstein College of Medicine, Bronx, New York, United States of America; 4 Department of Neuroscience, Albert Einstein College of Medicine, Bronx, New York, United States of America; The Ohio State University, United States of America

## Abstract

The proteasome cleaves intracellular proteins into peptides. Earlier studies found that treatment of human embryonic kidney 293T (HEK293T) cells with epoxomicin (an irreversible proteasome inhibitor) generally caused a decrease in levels of intracellular peptides. However, bortezomib (an antitumor drug and proteasome inhibitor) caused an unexpected increase in the levels of most intracellular peptides in HEK293T and SH-SY5Y cells. To address this apparent paradox, quantitative peptidomics was used to study the effect of a variety of other proteasome inhibitors on peptide levels in HEK293T and SH-SY5Y cells. Inhibitors tested included carfilzomib, MG132, MG262, MLN2238, AM114, and clasto-Lactacystin β-lactone. Only MG262 caused a substantial elevation in peptide levels that was comparable to the effect of bortezomib, although carfilzomib and MLN2238 elevated the levels of some peptides. To explore off-target effects, the proteosome inhibitors were tested with various cellular peptidases. Bortezomib did not inhibit tripeptidyl peptidase 2 and only weakly inhibited cellular aminopeptidase activity, as did some of the other proteasome inhibitors. However, potent inhibitors of tripeptidyl peptidase 2 (butabindide) and cellular aminopeptidases (bestatin) did not substantially alter the peptidome, indicating that the increase in peptide levels due to proteasome inhibitors is not a result of peptidase inhibition. Although we cannot exclude other possibilities, we presume that the paradoxical increase in peptide levels upon treatment with bortezomib and other inhibitors is the result of allosteric effects of these compounds on the proteasome. Because intracellular peptides are likely to be functional, it is possible that some of the physiologic effects of bortezomib and carfilzomib arise from the perturbation of peptide levels inside the cell.

## Introduction

**Figure 1:**
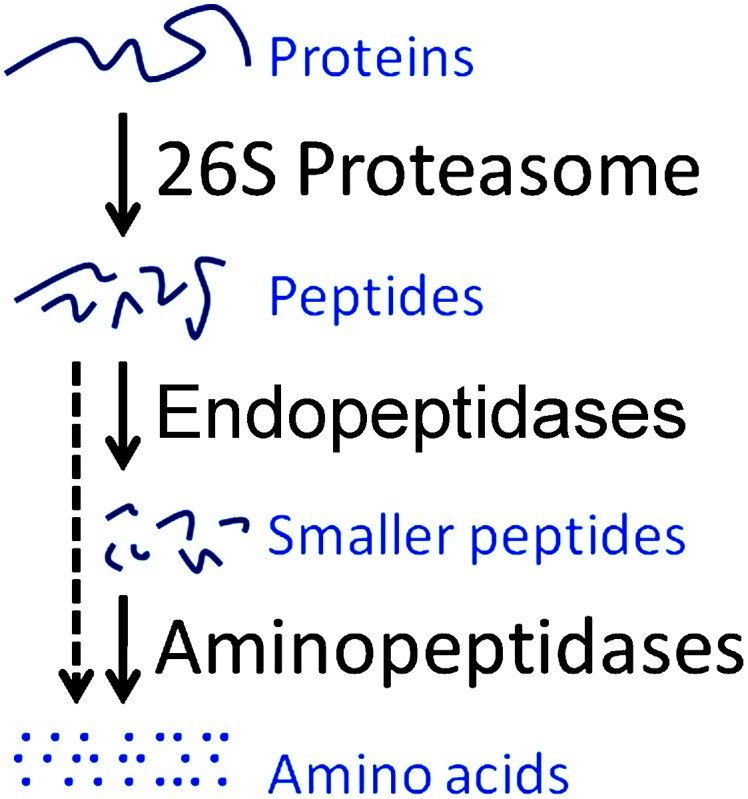
Cytoplasmic protein turnover. The 26S proteasome, a multicatalytic complex cleaves proteins into peptides typically 3–25 residues long, which are further degraded into amino acids by a variety of downstream endopeptidases and/or aminopeptidases.

A major pathway of intracellular protein degradation involves the proteasome, a multi-subunit enzyme complex that resides in the cytosol and nucleus [1], [2]. Proteins destined for degradation, usually by the covalent addition of ubiquitin, are transported into the interior of the proteasome where they encounter the active protease subunits. There are three active subunits: beta 1 (also referred to as caspase-like); beta 2 (referred to as trypsin-like); and beta 5 (referred to as chymotrypsin-like). The proteasome cleaves proteins into peptides typically 3–25 residues long [3], and these peptides are usually further degraded into amino acids by a variety of cellular enzymes such as oligoendopeptidases, tripeptidyl peptidase 2 (TPP2), and aminopeptidases [4]–[9] (Figure 1). A small percentage of the peptides produced by the proteasome are transported into the endoplasmic reticulum and incorporated into major histocompatibility complex (MHC) class I proteins, which present the peptides on the cell surface [10]. Although many proteasome degradation products are rapidly destroyed by aminopeptidases [11], mass spectrometry based peptidomic studies detected a large number of protein-derived peptides in animal tissues and cell lines [12], [13]. Only a small portion of the peptides detected in the peptidomic studies were derived from the most abundant or most unstable cellular proteins, suggesting that these peptides did not merely reflect protein turnover [13]. Recently, several studies have found that intracellular peptides are functional and influence signal transduction as well as other cellular processes [14]–[17].

In an effort to identify the source of the intracellular peptides, previous studies treated SH-SY5Y cells (a human neuroblastoma cell line) and/or HEK293T cells (a human embryonic kidney cell line) with proteasome inhibitors and examined the effect on the cellular peptidome [18], [19]. One study involved the proteasome inhibitor epoxomicin, an irreversible inhibitor that potently blocks the beta 5 site and also inhibits the beta 2 site at higher concentrations [19]. Most, although not all of the peptides that required cleavage at hydrophobic sites were reduced by treatment with either low (0.2 µM) or high (2 µM) concentrations of epoxomicin, consistent with the hypothesis that the proteasome was responsible for production of these peptides. In the absence of the proteasome inhibitor, low levels of peptides arising from cleavage at beta 1 sites were detected in the cellular peptidome, but when cells were treated for 1 hour with 2 µM epoxomicin, the levels of these peptides were greatly elevated. This is consistent with the concept that once proteins are transported into the proteasome, they cannot exit until degraded and if the preferred enzyme (i.e. the beta 5 subunit) is not active, then cleavage by the other subunits becomes the primary route of degradation.

Another previous study examined the effect of bortezomib on the cellular peptidome [18]. Bortezomib is a reversible proteasome inhibitor containing an active site boronate group and is FDA-approved to treat multiple myeloma and mantle cell lymphoma. Bortezomib is a potent inhibitor of the beta 5 subunit, and at higher concentrations blocks the beta 1 subunit [20]. Because the beta 5 subunit plays a major role in the conversion of proteins into peptides, and bortezomib potently inhibits this subunit, it was expected that this drug would cause a decrease in the levels of these peptides, as found for epoxomicin [19]. However, the opposite result was found; the majority of intracellular peptides was elevated by treatment with bortezomib, including many peptides that were predicted to be products of beta 5 cleavages [18]. One possible explanation of this paradoxical result is that bortezomib has off-target effects on the enzymes that degrade the intracellular peptides (Figure 1); a previous study predicted that bortezomib may inhibit TPP2, based on the finding that bortezomib inhibited other cellular serine proteases such as cathepsins A and G [21]. Alternatively, bortezomib is known to allosterically influence proteasome stability, gate opening, and cleavage specificity [22]–[26], and it is possible that these allosteric effects cause the increase in cellular peptides upon exposure to bortezomib.

To study this, we used a peptidomics method to examine the effect of a variety of proteasome inhibitors on the peptidome of HEK293T and SH-SY5Y cells; these cell lines were used because their peptidomes have been well-studied. The inhibitors picked for this analysis include three boronate compounds that inhibit the proteasome reversibly (MG262, MLN2238, and AM114), and three non-boronate compounds, one of which is an irreversible inhibitor (clasto-Lactacystin β-lactone) and two of which are reversible inhibitors (MG132 and carfilzomib) [27]. Carfilzomib is an analog of epoxomicin that was recently approved for the treatment of multiple myeloma and mantle cell lymphoma [21], [28]. Some of these proteasome inhibitors are known to have off-target effects, such as MG132 which inhibits calpain and clasto-Lactacystin β-lactone which inhibits cathepsin A [29]. We also tested bortezomib as an inhibitor of peptidases present in HEK293T cells using assays that detect TPP2 and puromycin-sensitive aminopeptidase. Finally, we examined whether potent inhibitors of these two enzymes influenced the peptidome of HEK293T cells. Although bortezomib, MG262, and one of the other boronate-containing proteasome inhibitors (MLN2238) are weak inhibitors of HEK293T cell aminopeptidase activity, this effect does not appear to contribute to the large increase in most cellular peptides observed with bortezomib and MG262, and to a lesser extent, with carfilzomib.

## Materials and Methods

### Materials

High glucose Dulbecco’s Modified Eagle’s Medium (DMEM), L-glutamine enriched Roswell Park Memorial Institute medium (RPMI) and Dulbecco’s Phosphate Buffered Saline (DPBS) were purchased from Invitrogen. Hydroxylamine, glycine, sodium hydroxide, dibasic sodium phosphate and dimethyl sulfoxide (DMSO) were obtained from Sigma. Acetonitrile was obtained from Fisher. Hydrochloric acid, trifluoroacetic acid (TFA) mass spectroscopy grade and C-18 spin columns were purchased from Pierce Thermo Scientific. MG132, MG262 and clasto-Lactacystin β-lactone were purchased from Boston Biochem. AM114, butabindide and puromycin were purchased from Tocris Bioscience. Other inhibitors and their commercial sources were bortezomib (LC Laboratories), MLN2238 (Selleckchem), carfilzomib (ChemieTek), bestatin (Sigma), and bestatin methylester (Calbiochem). Recombinant human puromycin-sensitive aminopeptidase was purchased from R&D Systems. Suc-Leu-Leu-Val-Tyr-AMC, Ala-Ala-Phe-AMC, Leu-AMC and Ala-AMC were procured from Bachem. The isotopic labeling reagents 4-trimethylammoniumbutyryl-N-hydroxysuccinimide (TMAB-NHS) containing either 0, 3, 6, or 9 atoms of deuterium (D0-, D3-, D6-, and D9-TMAB-NHS, respectively) or 9 atoms of deuterium and three ^13^C atoms (D12-TMAB-NHS) were synthesized as described [30].

### Methods

#### Large scale cell culture, treatment with proteasomal and aminopeptidase inhibitors, peptide extraction

HEK293T and SH-SY5Y cells were grown to 80–90% confluence in DMEM and RPMI supplemented with 10% fetal bovine serum and penicillin/streptomycin. A single plate of cells (150 mm×25 mm) was used for each group. At the start of the experiment, media were removed from the plates and cells were washed with DPBS (138 mM NaCl, 8.06 mM Na_2_HPO_4_, 2.67 mM KCl, 1.47 mM KH_2_PO_4_, 0.9 mM CaCl_2_ and 0.49 mM MgCl_2_). This was followed by addition of serum-free media containing the proteasome or aminopeptidase inhibitors, or 0.05% DMSO as a control for the inhibitors that were soluble in DMSO. Each experiment consisted of two control groups and two or three replicate groups of cells treated with one of the inhibitors. The cells were incubated at 37°C for 20 minutes, following which media containing the inhibitor was removed, cells were washed with DPBS, detached from the plate by scraping into DPBS (without trypsin), and centrifuged at 800×*g* for 5 min. The wash buffer was supplemented with the appropriate inhibitor at the same concentration as used for the treatment, thereby ensuring that the changes induced in peptide levels were not reversed during harvesting of cells due to possible dissociation of the reversible inhibitors. The wash time (including centrifugation) was 15 minutes, for a total treatment time of 35 minutes. After centrifugation, the cell pellet was resuspended in 1 ml of 80°C water and incubated in an 80°C water bath for 20 minutes to inactivate proteases. The mixture was again centrifuged (13,000×*g*, 30 min, 4°C) and was stored at −80°C overnight. For peptide extraction, the samples were thawed and centrifuged again. The lysate was subsequently cooled on ice and acidified with HCl to a final concentration of 10 mM. After 15 min incubation on ice, the lysate was centrifuged at 13,000×*g* for 30 min at 4°C. To the supernatant, 250 µL of dibasic sodium phosphate (0.4 M, pH 9.5) was added and the mixture was stored at −80°C until labeling.

#### Isotopic labeling and mass spectrometry

The labeling protocol has been earlier described in detail [30]. Each group within an experiment (control/treated) was labeled with a different isotopic tag. The TMAB-NHS labels were dissolved in DMSO to a concentration of 400 µg/µL and 15 mg of label was used per 150 mm plate of cells. Typically, 1.5 mg of protein is obtained from each 150 mm plate of cells. At the beginning, the pH of the peptide extract was adjusted to 9.5 with 1 M NaOH. Labeling was performed over 8 rounds; 4.69 µL of the label was added to the extract every 20 min. The pH was measured between each round and if necessary, brought back to 9.5 for the first five rounds. For rounds 6–8, the pH was not adjusted after the addition of the TMAB-NHS reagent. After the final round of label addition, the pH was adjusted to 9.5 again and the extracts were incubated at room temperature for 90 min. Thereafter, 30 µL of 2.5 M glycine was added to quench any unreacted label. Following 40 min incubation at room temperature, the labeled extracts for a single experiment were pooled and filtered through Amicon Ultracel-10 K units. It is important to ensure that only N-terminal amines and lysine side-chain amines of peptides are TMAB-labeled and not tyrosines. To hydrolyze any labeled tyrosine, 30 µL of a 2 M solution of hydroxylamine hydrochloride (pH 3.7) was added over three rounds to the pooled and filtered sample. The pH was measured after the addition of hydroxylamine and adjusted to 9.0 with 1 M NaOH. The samples were desalted through PepClean C-18 spin columns (Thermo Scientific, Rockford, IL, USA) by following manufacturer’s instructions. Peptides were eluted using 160 µL of 0.5% TFA and 70% acetonitrile, frozen at −80°C and then lyophilized in a vacuum centrifuge and stored at −80°C until analysis by mass spectrometry.

The LC-MS/MS experiments were performed on a Synapt G2 mass spectrometer coupled to a NanoAcquity capillary liquid chromatography system (Waters Co., Milford, MA, USA). The peptide mixture was desalted online for 3 min at a flow rate of 5 µL/min using a Symmetry C18 trapping column (5-µm particles, 180-µm inner diameters, 20-mm length; Waters Co., Milford, MA, USA). The mixture of trapped peptides was subsequently separated by elution with a water/acetonitrile/0.1% formic acid gradient through a BEH 130-c18 column (1.7-µm particles, 100-µm inner diameters, 100-mm length; Waters Co., Milford, MA, USA). The data were acquired in the data-dependent mode, and the multiple-charged protonated peptides generated by electrospray ionization (ESI) were automatically mass selected and dissociated in MS/MS by 15- to 60-eV collisions with argon. The typical LC and ESI conditions consisted of a flow rate of 600 nL/min, a capillary voltage of 3.5 kV, a block temperature of 100°C, and a cone voltage of 100 V.

MS spectra were analyzed using the MassLynx software (Waters). Peak groups representing peptides labeled with different isotopic labels were identified and the relative intensity of each isotopic peak was determined using both the monoisotopic and the peak containing one ^13^C atom and subtracting baseline noise. To quantify relative peptide levels, the peak intensity of each treated group was compared to the average of the control replicates in each experiment.

To identify peptides, MS/MS data were analyzed using the Mascot search engine (Matrix Science Ltd, UK) and the IPI_human data base (91,464 sequences; 36,355,611 residues). Searches include variable modifications of N-terminal acetylation, methionine oxidation, and the isotopic D0-, D3-, D6-, and D9-TMAB tags used in our study (listed on Mascot as GIST-Quat). Results were manually interpreted to eliminate false positives, using previously described criteria [18], [30]–[33].

#### Enzyme assays

All enzyme assays described below were performed in the linear range of the assay, as determined from time-course studies. HEK293T cells were lysed by sonication in 50 mM TrisHCl buffer, pH 7.5, containing 40 mM KCl, 5 mM MgCl2, 0.5 mM ATP, and 1 mM DTT (Buffer 1).

To measure proteasome activity, a 1∶50 dilution of the cell extract was pre-incubated with the inhibitor and Buffer 1 for 30 min, followed by addition of 100 µM final concentration of the proteasome substrate succinyl-Leu-Leu-Val-Tyr-7-amino-4-methylcoumarin (Suc-Leu-Leu-Val-Tyr-AMC). After incubation at 37°C for 1 hour, proteasomal activity was quantified by fluorescence measurement of the substrate (380 nm excitation, 460 nm emission).

To measure TPP2, the fluorescent substrate was Ala-Ala-Phe-AMC. Aminopeptidase (AP) assays were performed with Leu-AMC and Ala-AMC, which detect leucine aminopeptidase (LAP), puromycin-sensitive aminopeptidase (PSAP), and other enzymes. To measure the effect of aminopeptidase/proteasome inhibitors on cleavage of Ala-Ala-Phe-AMC and Leu-AMC, 1∶50 dilution of the cell extract was pre-incubated with the inhibitor and Buffer 1 for 30 min, followed by addition of 100 µM final concentration of the respective substrate. For Ala-AMC, the same dilution of the cell extract was pre-incubated with the inhibitor and 50 mM MOPS buffer, pH 7.5, containing 40 mM KCl, 5 mM MgCl2, 0.5 mM ATP, and 1 mM DTT (Buffer 2), followed by addition of 100 µM final concentration of the substrate. After incubation at 37°C for 1 hour, enzyme activity was quantified by fluorescence measurement of the substrate (380 nm excitation, 460 nm emission).

To measure the effect of inhibitors on cleavage of Leu-AMC and Ala-AMC by purified PSAP, recombinant human PSAP (rhNPEPPS, R&D Systems) was diluted 1∶2000 in Buffer 2 containing 0.1 mg/ml BSA. The enzyme was pre-incubated with the inhibitor and Buffers 1 or 2, followed by addition of 100 µM final concentration of Leu-AMC (to the mix containing Buffer 1) and Ala-AMC (to the mix containing Buffer 2). After incubation at 37°C for 1 hour, PSAP activity was quantified by fluorescence measurement of the substrate (380 nm excitation, 460 nm emission).

The effect of bortezomib on the chymotryptic-like activity of different forms of the proteasome from yeast were performed as described previously [34], with minor modifications. Briefly, purified 20S subunit, Blm10-20S, open gate mutant of the 20S subunit and 26S (each containing 1 nM of the 20S subunit) were incubated in 50 mM TrisHCl buffer, pH 7.5 containing 5 mM MgCl_2_, 0.5 mM EDTA and 100 µM Suc-Leu-Leu-Val-Tyr-AMC with or without 1 mM ATP and bortezomib for 15 minutes at 30°C. Proteasome activity was quantified by measuring the kinetics of fluorescence release (380 nm excitation, 460 nm emission). Fluorescence was measured at an interval of 30 seconds for 30 minutes with 5 seconds of mixing prior to each cycle. Proteasomes were purified as described previously [34], [35].

## Results

**Figure 2:**
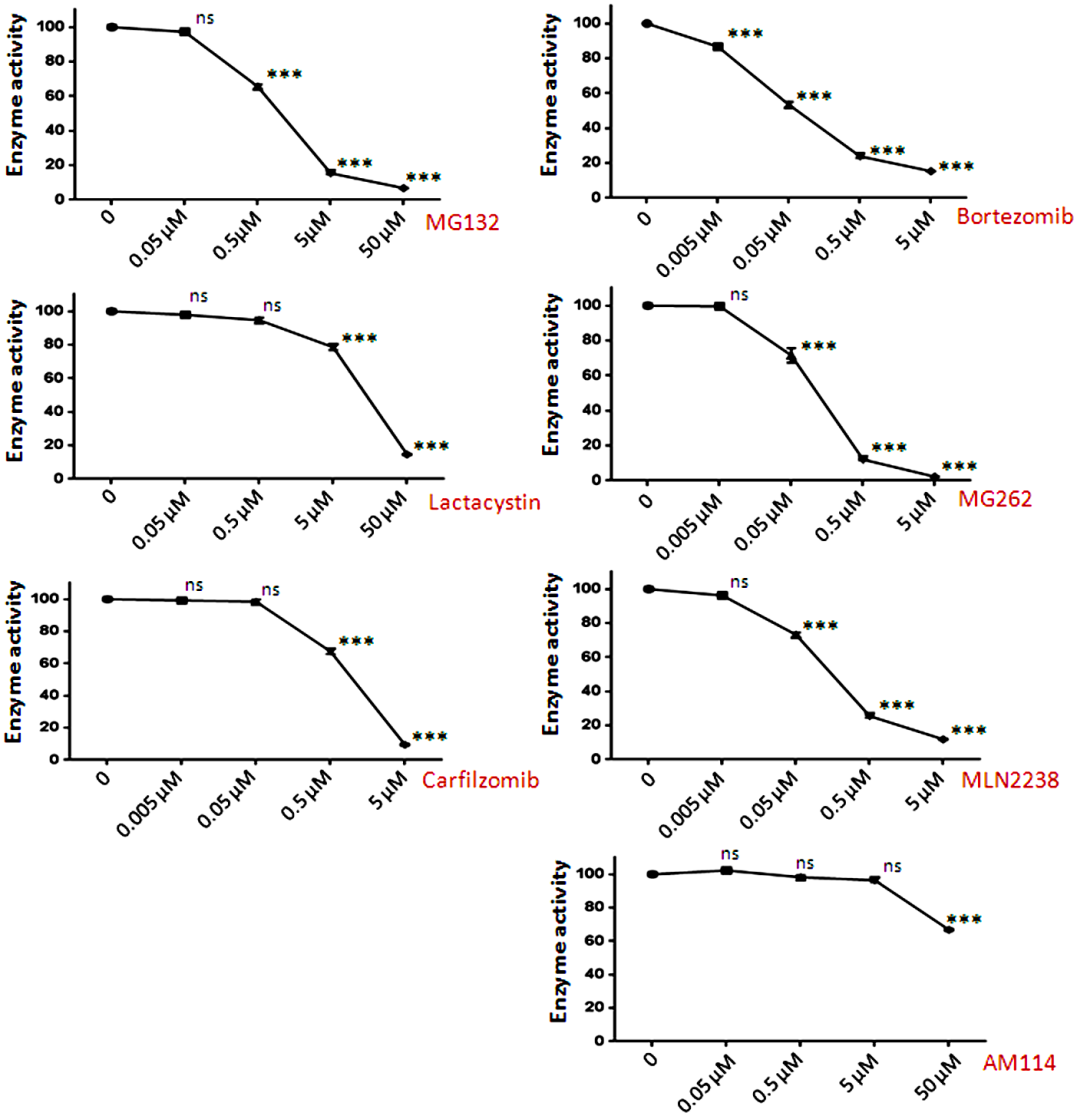
Inhibition of proteasome activity in HEK293T cells with various proteasome inhibitors. HEK293T cell extract was treated with increasing concentrations of the indicated compound for 30-Leu-Leu-Val-Tyr-AMC and incubation for 1 hour at room temperature. Enzyme activity was determined by fluorescence measurement of AMC. The resulting activity is expressed as percent enzyme activity relative to the control reaction without inhibitor. The error bars show standard error of mean (n = 3); points without error bars had error ranges smaller than the symbol size. Statistical analysis was performed using Student’s t-test: ***, p≤0.001; ns, no significant difference (p>0.05) versus the control without inhibitor.

To investigate the discrepancy between the effect of epoxomicin and bortezomib on the levels of intracellular peptides, we tested six additional proteasome inhibitors. All compounds were first evaluated for their ability to inhibit proteasome activity in HEK293T cell extracts, assayed using the beta 5-selective substrate Suc-Leu-Leu-Val-Tyr-AMC. Two of the compounds displayed IC_50_ values of 100–200 nM (MG262 and MLN2238), which is comparable to that observed with bortezomib (Figure 2). MG132 and carfilzomib had IC_50_ values around 1 µM, clasto-Lactacystin β-lactone had an IC_50_ of ∼10 µM, and AM114 was approximately 10-fold less potent and did not produce more than 35% inhibition at the highest concentration tested (Figure 2); these values are in general agreement with previous reports [20], [28], [29], [36], [37]. Because AM114 contains two boronate groups, it was included in subsequent studies to test if the previously observed effects of bortezomib were caused by off-target effects due to the boronate groups rather than inhibition of the proteasome.

**Figure 3:**
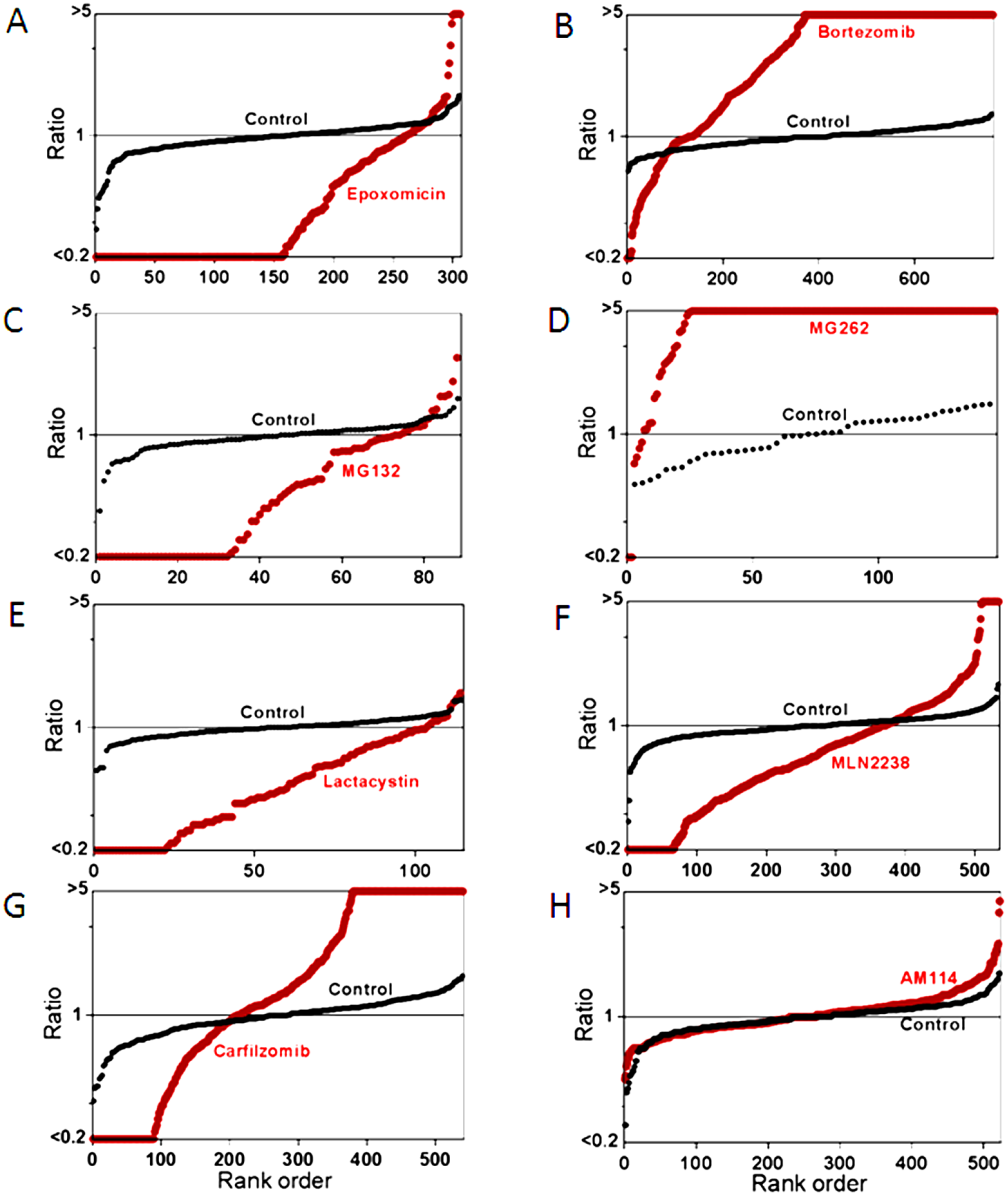
Summary plots of the peptidome of HEK293T cells in response to proteasome inhibitors. The relative level of all the peptides identified by MS/MS analysis in each of the drug-treated replicates was compared to the average level of the peptide in the untreated control replicates. The y-axis represents the relative peptide levels (log-scale) sorted from low to high and the x-axis represents the rank order of each peptide sorted according to the relative level. When a peptide is detected in multiple charged states, the peptide ratio for each peptide is indicated separately. If the ratio was <0.20 or >5.0 between the drug-treated and control replicates, the value was capped at 0.20 or 5.0. Each panel shows two plots; one shows the peptide level of each replicate of the identified peptides in drug-treated cells, expressed relative to the average control value (red circles), the other shows the peptide level of each control replicate expressed relative to the average control value (black circles). Panels A–H represent data from experiments on HEK 293T cells treated with (A) 0.2 µM epoxomicin for 1 hour, (B) 0.5 µM bortezomib for 1 hour, (C) 2.5 µM MG132 for 35 min, (D) 1 µM MG262 for 35 min, (E) 1 µM clasto-Lactacystin β-lactone for 35 min, (F) 1 µM MLN2238 for 35 min, (G) 1 µM carfilzomib for 35 min, (H) 1 µM AM114 for 35 min. The data used to generate the plots shown in panels A and B were previously published [18], [19].

**Figure 4:**
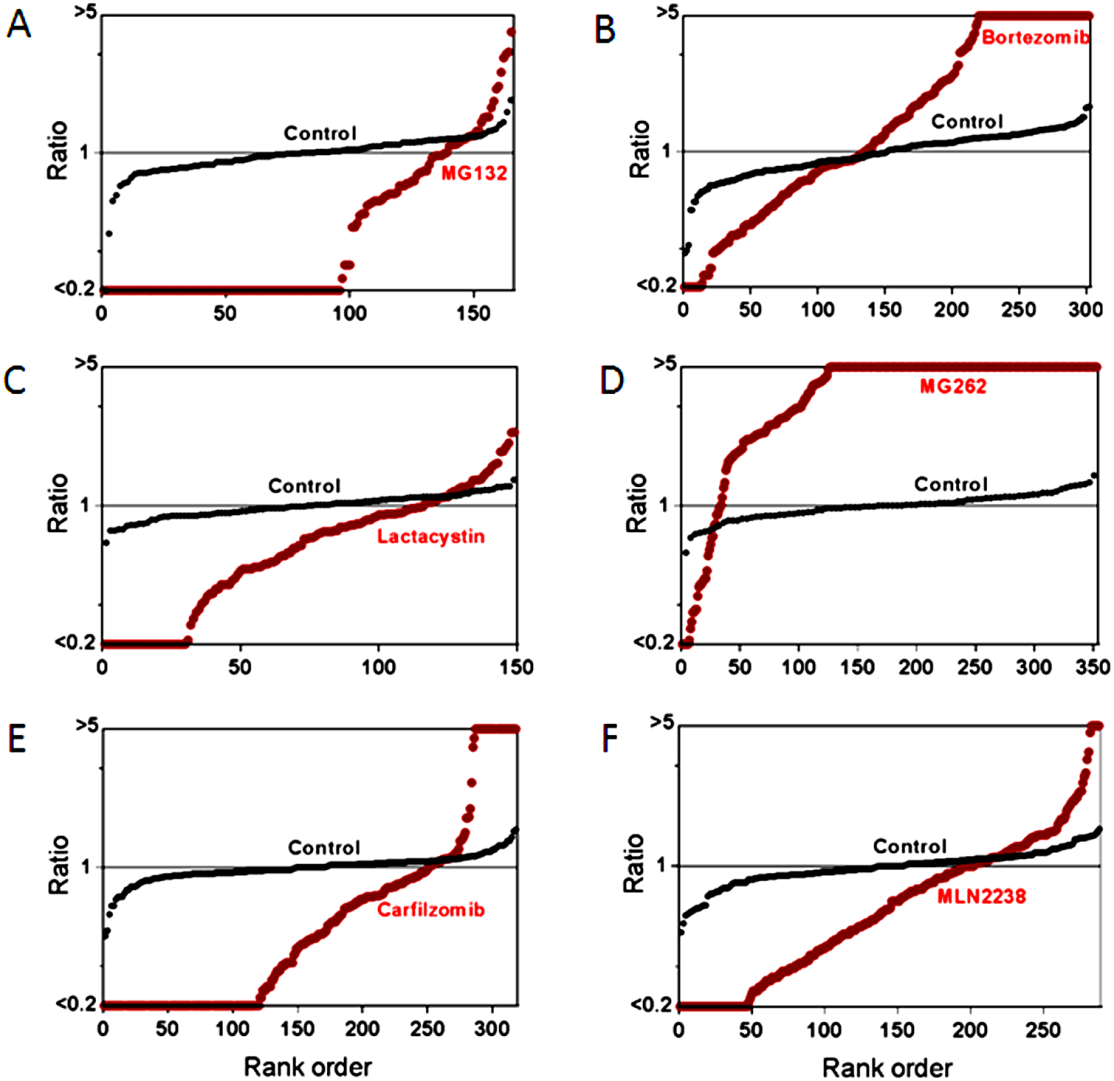
Summary plots of the peptidome of SH-SY5Y cells in response to proteasome inhibitors. Panels A–F represent data from experiments on SH-SY5Y cells treated with (A) 2.5 µM MG132 for 35 min, (B) 0.5 µM bortezomib for 1 hour, (C) 1 µM clasto-Lactacystin β-lactone for 35 min, (D) 1 µM MG262 for 35 min, (E) 1 µM carfilzomib for 35 min, (F) 1 µM MLN2238 for 35 min. Specifications and criteria for making the summary plots are described in Figure 3. The data used to generate the plot shown in panel B was previously published [18].

The previous study investigating the effect of bortezomib on the cellular peptidome found generally similar results for treatment times ranging from 30–90 minutes [18]. In the present study, a short treatment time (35 minutes) was used to reduce the contribution from secondary changes due to altered protein levels, cell stress, or cell death; these do not occur upon short exposure of cells to proteasome inhibitors. Peptide levels were measured using a quantitative peptidomics technique that uses stable isotopic labels to compare up to five samples in a single experiment [30]. For all of these analyses, 2–3 replicates of inhibitor-treated cells were compared to 2 replicates of control cells (incubated with comparable concentration of DMSO in the absence of inhibitor). Relative levels of peptides were quantified by measurement of peak height for each of the isotopic peaks detected in the MS spectra, and peptides were subsequently identified by MS/MS analysis using rigorous criteria previously established for peptidomics [30], [38], [39]. Because the peptide levels are expressed as a relative ratio, any peptide not detected in one of the groups of replicates was capped at a level 1/5^th^ that of the observed peptide; this means that peptides only detected in the control groups and not in the treated samples are listed with ratios ≤0.20 while those found only in the treated groups are listed with ratios of ≥5 (Table S1). In addition to including all data in a supplementary file (Table S1), the results are graphically represented in rank order plots (Figures 3 and 4). To generate these plots, the ratio of the level of peptide in each of the biological replicates was compared to the average level in the control replicates and then sorted by rank order and plotted. The y-axis represents the relative level of peptide in the indicated replicate (either inhibitor-treated or control replicates) and the x-axis is the rank order of the peptides. In most of the control replicates, each individual replicate did not differ by more than 2-fold from the average of the two controls (i.e. ratios between 0.5 and 2.0), with an average ratio of 1.0 (Figures 3 and 4). In contrast, very few of the peptides in the inhibitor-treated groups had ratios around 1.0, and most peptides were either much higher or lower than this ratio (except for the cells treated with AM114, which were similar to the control group).

Treatment of HEK293T cells with MG132, clasto-Lactacystin β-lactone, or MLN2238 produced changes in the peptidome that were generally similar to those caused by the treatment with 0.2 µM epoxomicin (Figure 3); the majority of peptides was greatly decreased by the proteasome inhibitor and few peptides were elevated. Similar changes were observed with MG132, clasto-Lactacystin β-lactone, and MLN2238 when tested with SH-SY5Y cells (Figure 4). In contrast, treatment of the cells with MG262 produced changes that were generally similar to those caused by 500 nM bortezomib (Figures 3 and 4), which were also similar to those produced by 50 nM bortezomib [18]. Carfilzomib decreased the levels of many peptides but also elevated levels of a number of other peptides in HEK293T cells (Figure 3) and SH-SY5Y cells (Figure 4). Because AM114 did not produce a substantial change in levels of peptides in HEK293T cells (Figure 3) and did not substantially inhibit the proteasome (Figure 2), this compound was not further tested in SH-SY5Y cells.

**Figure 5:**
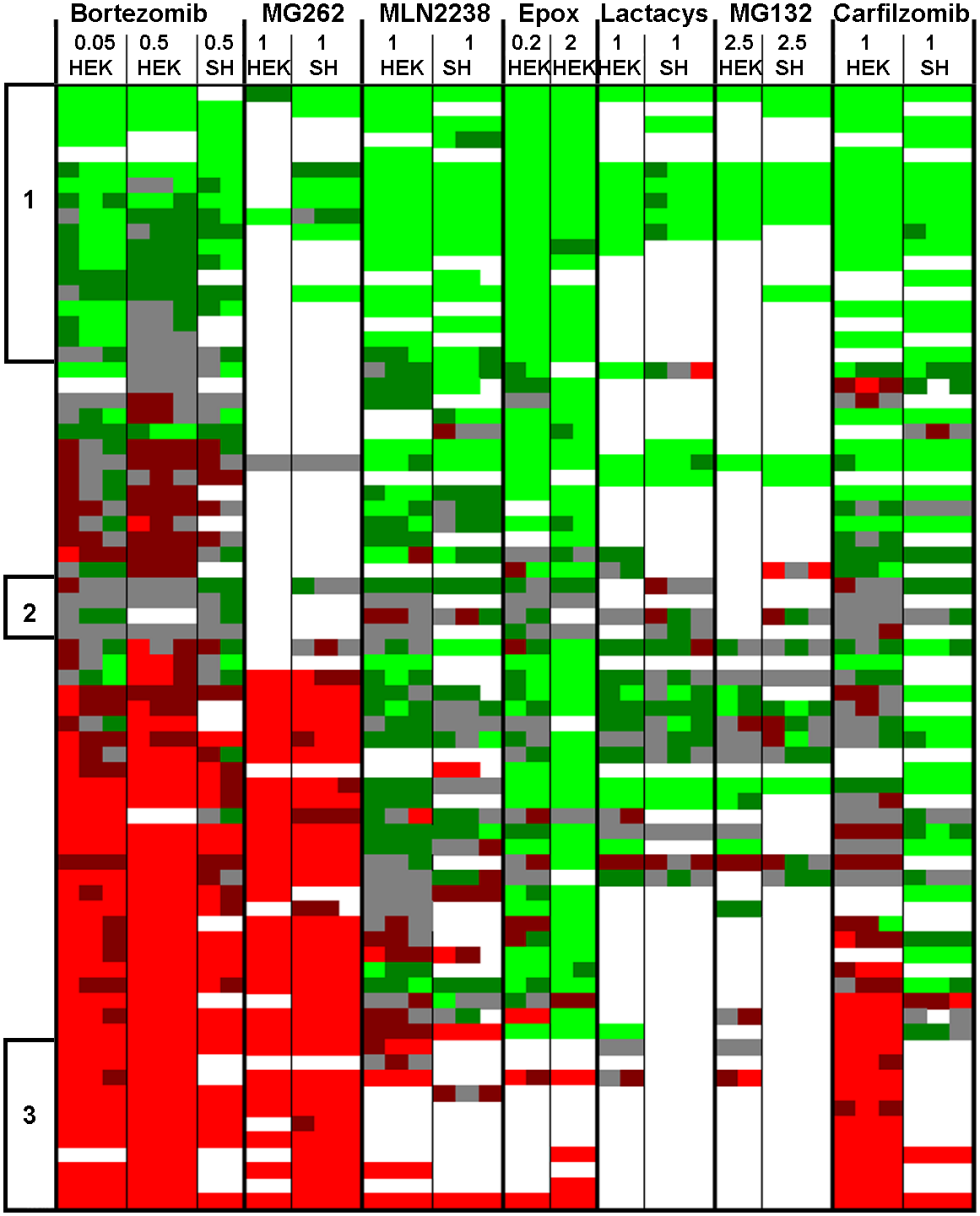
Heat map analysis of selected peptides. Peptides observed in multiple experiments were chosen for this analysis. Different peptides are plotted as separate rows; each column indicates a different experiment and each sub-column represents peptide levels in the biological replicates within that experiment. Ratios of peptides found in multiple charged states were averaged together. White panels could not be accurately quantified, either due to undetectable signals or peak overlap with another co-eluting peptide. Data from the AM114 experiment was not included in the heat map since levels of most peptides were not affected by AM114. Peptide levels were color coded. Bright green indicates peptides that were present in the treated cells at levels ≤50% of those in the control cells (ratio ≤0.50). Dark green indicates peptides that showed a smaller decrease (ratio 0.51–0.80). Grey indicates peptides that were close to the levels in the control cells (ratio 0.81–1.24). Dark red indicates peptides that were elevated 25–99% (ratio 1.25–1.99). Bright red indicates peptides that were elevated ≥100% (ratio ≥2.00). The three groups in the heat map indicated by lines and numbers on the left margin indicate peptides which showed a large or partial decrease (set 1), little or no change (set 2), or a large increase (set 3) in response to the proteasome inhibitors. See Table S2 for an expanded version of this figure, including peptide sequences and ratio values.

While the summary plots shown in Figures 3 and 4 provide a visual representation of the overall pattern of peptide levels, these plots do not provide information about specific peptides. Table S1 contains data on every peptide detected in each experiment, both identified and unknowns, but due to the size of this table it is difficult to compare trends among different peptides. To compare levels of specific peptides between datasets, heat maps were created (Figure 5). For these analyses, peptides that were found in multiple experiments were placed into a single table and the relative levels of peptide in each of the experimental replicates were color-coded, with green indicating peptides that were decreased in the treated cells, red indicating peptides that were elevated in the treated cells, gray indicating peptides that were not greatly affected by the treatment, and missing data in white. Table S2 shows the data with values and peptides sequences, while Figure 5 shows only the color-coded results. To facilitate comparison of the new data with previous results using different proteasome inhibitors, the heat maps include previously reported data for bortezomib (HEK293T cells treated with 50 nM or 500 nM for 1 hour, or SH-SY5Y cells treated with 500 nM for 1 hour) and epoxomicin (HEK293T cells treated with 0.2 or 2 µM for 1 hour). The heat map analysis shown in Figure 5 excludes AM114, which did not substantially inhibit the proteasome at the concentration used in the peptidomics study (the data with AM114 are included in Table S2). Each biological replicate within an experiment is indicated as a separate column, allowing for variability of each peptide among replicates to be compared (Figure 5).

Peptides selected for inclusion in the heat map were chosen based on the number of times found in each of the distinct experiments; peptides found in at least five separate runs are included. Only a handful of peptides were detected in every replicate in every experiment. The failure to detect a peptide doesn’t necessarily mean it isn’t present; there are multiple reasons for the absence of a signal. In general, the studies with MG132, MG262, and clasto-Lactacystin β-lactone resulted in fewer detectable peptides than the other studies. Despite this limitation, several trends were detected in the heat map analysis. First, many of the same peptides elevated upon treatment of cells with bortezomib are also elevated by MG262 (Figure 5). In contrast, most of the other proteasome inhibitors do not cause these peptides to be elevated (Figure 5). One exception is carfilzomib, which produces an increase in some but not all of the peptides elevated by bortezomib and MG262.

Another trend revealed by the heat map analysis is that some peptides show similar responses to all of the proteasome inhibitors. One set of peptides was decreased in at least five of the runs and had an average ratio in all runs of ≤0.65 (Figure 5, set 1). In some replicates, these peptides were in the “no change” group, but never showed an increase in any of the replicates. Altogether there were 18 peptides in this set (Table 1). The majority of these peptides represent the N-terminus or C-terminus of the protein, and therefore only a single cleavage is required to produce the peptide (in contrast to peptides that arise from internal sequences within the protein). All of the peptides in this set are produced by cleavages attributed to the beta 5 proteasome subunit, based on the presence of a hydrophobic amino acid residue in the P1 positions of the cleavage sites required to generate the peptide. Another set of peptides was not greatly affected by the proteasome inhibitors in any of the replicates (Table 2 and Figure 5, set 2). The average ratio for these peptides ranged from 0.85 to 0.99 in all of the studies, and in no case was a large change found in any of the replicates (i.e. no bright red or bright green in the heat map). One member of this group is the small protein thymosin beta-10, which only undergoes removal of the initiation methionine and would not be expected to be altered by treatment with proteasome inhibitors. A third set of peptides was found to increase in at least five of the experiments, with an average ratio >3.0 for all experiments, and no replicate showing a decrease in any of the experiments (Table 3 and Figure 5, set 3). Of the 11 peptides in this group, the majority represented internal fragments of the protein and therefore required two cleavages to be produced. Of the 18 cleavage sites required to produce these peptides, only 11 (61%) match the consensus site for beta 5 cleavages, the rest match the consensus site for beta 1 or beta 2 cleavages. There was no substantial difference in the average mass or peptide length for the peptides in set 1 versus set 3.

**Table 1:** Peptides decreased by treatment of cells with proteasome inhibitors.

Gene Name	Protein	P1	Peptide Sequence	P1′	Theor Mass
COX5A	Cytochrome c oxidase subunit 5a	l	GISTPEELGLDKV	*	1356.71
COX6B1	Cytochrome c oxidase subunit 6b	*m	Ac-AEDMETKIKNYKT	a	1611.78
EEF1B2	Eukaryotic translation elongation factor 1 beta	*m	GFGDLKSPAGLQVL	n	1400.77
NDUFA8	NADH dehydrogenase 1 alpha subcomplex, 8	*m	PGIVELPTLEEL	k	1308.72
NME2	Nucleoside diphosphate kinase B	*m	Ac-ANLERTFIAIKPDGV	q	1684.91
PARK7	Protein DJ-1 (Parkinson disease protein 7)	*m	Ac-ASKRALVIL	a	1011.63
PARK7	Protein DJ-1 (Parkinson disease protein 7)	*m	Ac-ASKRALVILA	k	1082.67
PEBP1	Phosphatidylethanolamine-binding protein 1	y	AGAAVDELGKVLTPTQV	k	1667.91
PFDN1	Prefoldin subunit 1	*m	Ac-AAPVDLELKKAFTEL	q	1685.92
PPIA	Peptidylprolyl isomerase A	l	KHTGPGILSM	a	1039.55
PPIA	Peptidylprolyl isomerase A	*m	VNPTVFFDI	a	1050.54
PPIA	Peptidylprolyl isomerase A	i	AVDGEPLGRVSF	e	1245.64
PPIA	Peptidylprolyl isomerase A	f	EDENFILKHTGPGILSM	a	1899.94
PRDX5	Peroxiredoxin 5	m	APIKVGDAIPAVEVF	e	1524.86
RBM3	RNA binding motif protein 3	*m	Ac-SSEEGKLFVGGLNF	n	1524.75
RPS21	40S Ribosomal protein S21	l	AKADGIVSKNF	*	1148.62
SRSF2	Splicing factor, arginine/serine-rich 2	*m	Ac-SYGRPPPDVEGMTSLKVDNL	t	2216.08
TPI1	Triosephosphate isomerase 1	l	ASQPDVDGFLVGGASLKPEFVDIINA	k	2658.35

Abbreviations: P1, amino acid residue in the P1 position of the cleavage site used to generate the N-terminus of the peptide; P1′, amino acid residue in the P1′ position of the cleavage site used to generate the C-terminus of the peptide; *, end of protein; *m, initiation methionine; Theor Mass, theoretical mass of the peptide; Ac-, acetyl. The amino acid sequence uses the standard single letter code and is indicated in upper case for the sequence of the peptide that was identified, and lower case for the peptide’s flanking sequences in the precursor protein.

**Table 2:** Peptides not altered substantially upon treatment of cells with proteasome inhibitors.

Gene Name	Protein	P1	Peptide Sequence	P1′	Theor Mass
COX7C	Cytochrome c oxidase subunit 7c	l	VVRHQLLKT	*	1092.68
PHB	Prohibitin	*m	Ac-AAKVFESIGKFGLA	l	1478.81
POMP	Proteasome maturation protein	*	Ac-MNARGLGSELKDSIPVTEL	s	2071.06
TMSB10	Thymosin beta-10	*m	Ac-ADKPDMGEIASFDKAKLKKTET QEKNTLPTKETIEQEKRSEIS	*	4933.52

Abbreviations are defined in Table 1.

**Table 3:** Peptides elevated by treatment of cells with proteasome inhibitors.

Gene Name	Protein	P1	Peptide Sequence	P1′	Theor Mass
FKBP1A	FK506 Binding Protein	d	VELLKLE	*	842.51
HSPE1	Heat shock 10 kDa protein 1 (chaperonin 10)	*m	Ac-AGQAFRKF	l	965.51
HSPE1	Heat shock 10 kDa protein 1 (chaperonin 10)	r	DGDILGKYVD	*	1093.53
HSPE1	Heat shock 10 kDa protein 1 (chaperonin 10)	k	GGIMLPEKSQGKVL	q	1455.81
HSPE1	Heat shock 10 kDa protein 1 (chaperonin 10)	k	GGIMLPEKSQGKVLQA	t	1654.91
NPM1	Nucleophosmin	q	ASIEKGGSLPKVEA	k	1384.76
NPM1	Nucleophosmin	m	TDQEAIQDLWQWRKSL	*	2016.01
RPLP2	60S acidic ribosomal protein P2	a	ALGGNSSPSAKDIKKI	l	1584.88
RPLP2	60S acidic ribosomal protein P2	i	LDSVGIEADDDRLNKVISE	l	2087.04
RPS28	40S Ribosomal protein S28	e	GDVLTLLE	s	858.47
TPI1	Triosephosphate isomerase 1	q	SLGELIGTLNAAKV	p	1384.79

Abbreviations are defined in Table 1.

**Figure 6:**
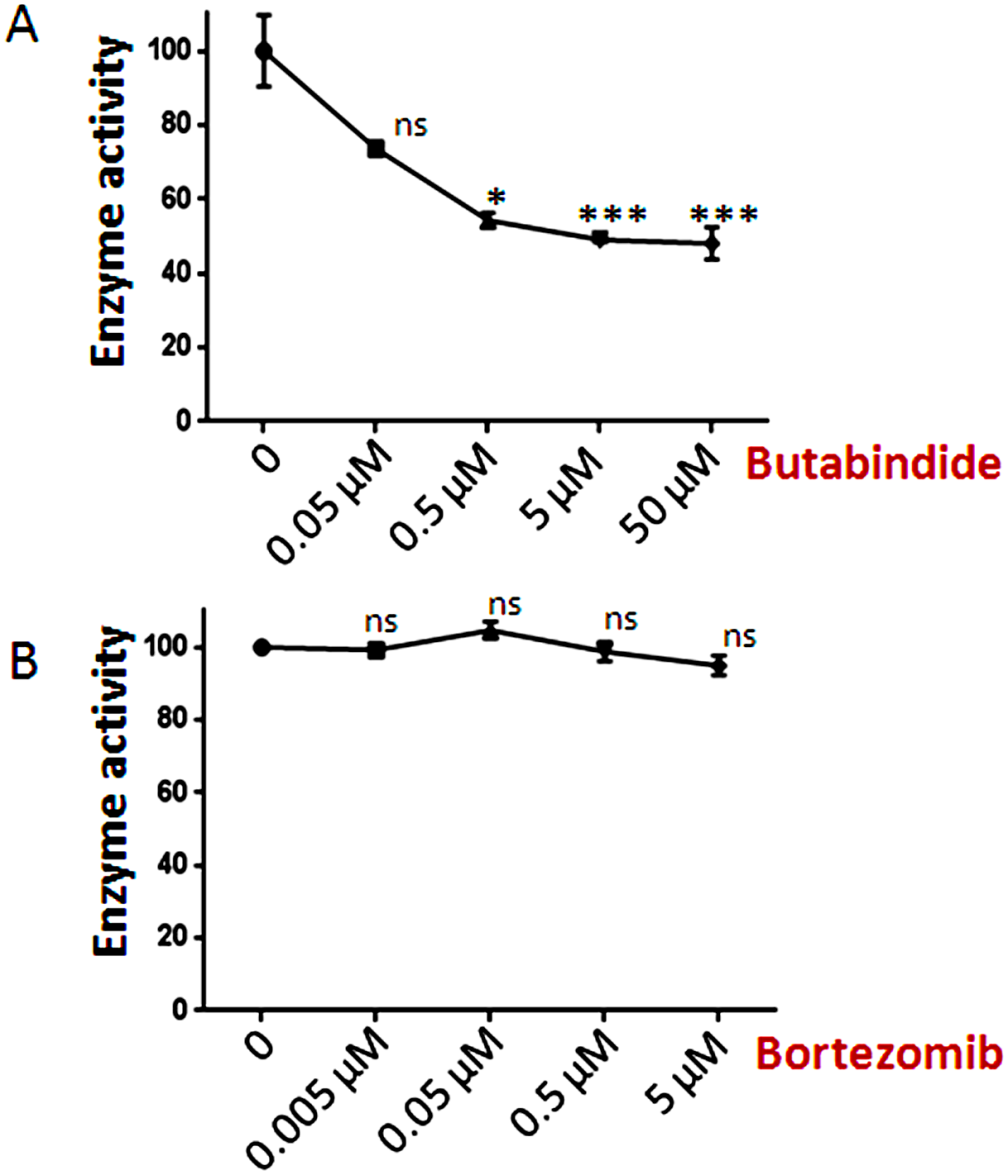
Effect of butabindide and bortezomib on Ala-Ala-Phe-AMC cleavage in HEK293T cells. HEK 293T cell extracts were treated with increasing concentrations of butabindide (Panel A) or bortezomib (Panel B) for 30 minutes in the absence of substrate, and then substrate was added and the reaction incubated for 1 hour. Enzyme activity was determined by fluorescence measurement of AMC and expressed as percentage enzyme activity relative to the control lacking inhibitor. Error bars show standard error of mean (n = 3), points without error bars have error ranges smaller than the symbol size. Statistical analysis was performed using Student’s t-test: *, p≤0.05; ***, p≤0.001; ns, no significant difference (p>0.05) versus the control.

The finding that bortezomib and other compounds increase the levels of some peptides can be explained by one of two possible mechanisms; either the compounds increase the formation of the peptides (possibly through allosteric activation of the proteasome) or the compounds block the degradation of the peptides (Figure 1). A recent study predicted that bortezomib could inhibit TPP2 [21]. TPP2 is thought to play a major role in peptide degradation within the cell [6], [40]. To test whether bortezomib inhibited TPP2, we first assayed HEK293T cell extracts with the TPP2 substrate Ala-Ala-Phe-AMC (Figure 6). Because this substrate is not specific for TPP2 and can be degraded by other cellular peptidases, we examined the activity in the presence of various concentrations of the TPP2-selective inhibitor butabindide. Approximately 50% of the Ala-Ala-Phe-AMC cleavage could be inhibited by micromolar concentrations of butabindide, suggesting that only half of the activity detected with this substrate was due to TPP2 (Figure 6, panel A). However, bortezomib did not show significant inhibition of the Ala-Ala-Phe-AMC cleavage, even at 5 µM concentrations, indicating that TPP2 is not substantially inhibited by bortezomib (Figure 6, panel B).

**Figure 7:**
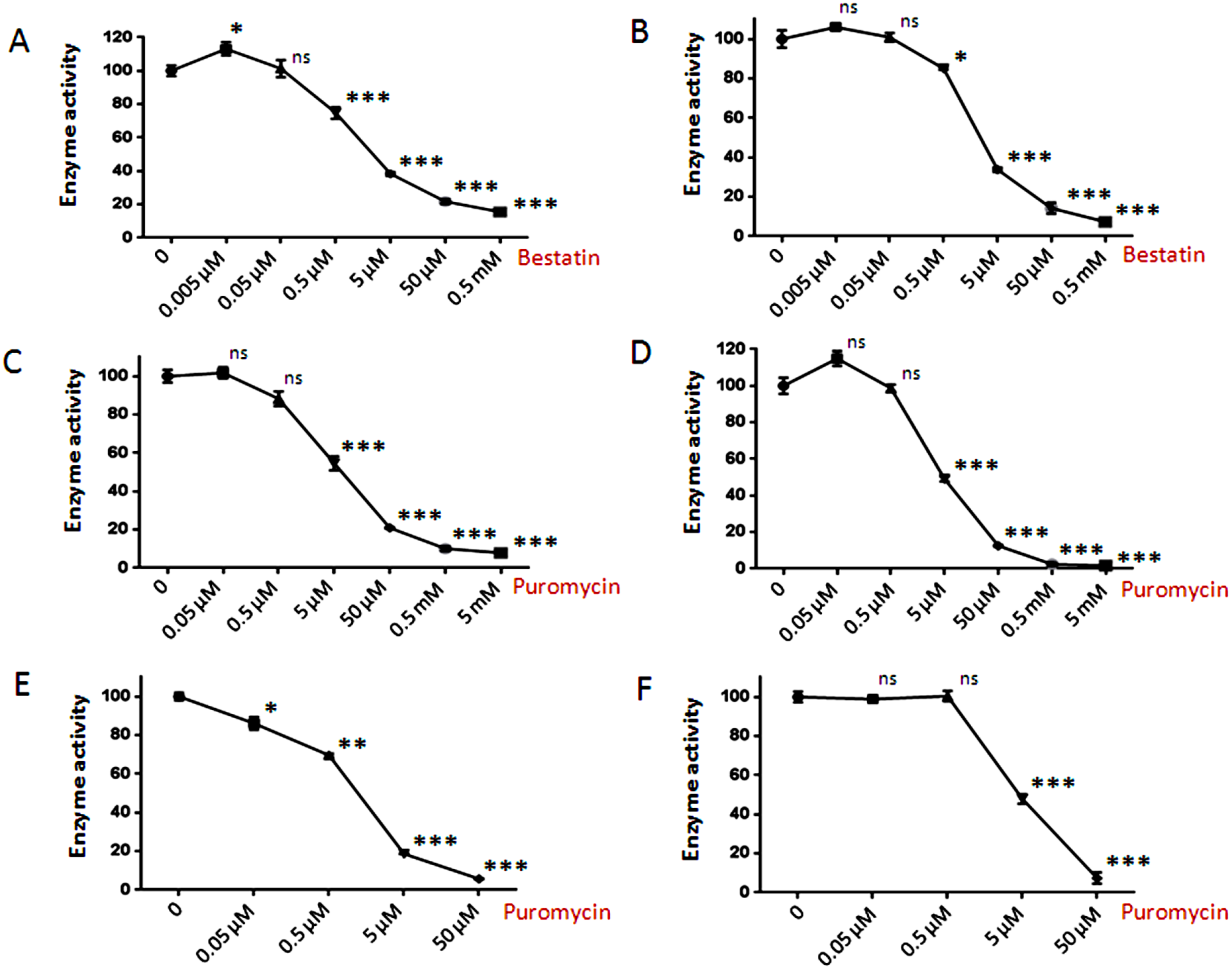
Effect of aminopeptidase inhibitors on Ala-AMC and Leu-AMC cleavage. HEK 293T cell extract (panels A–D) or recombinant PSAP (panels E–F) were treated with increasing concentrations of bestatin or puromycin for 30 minutes followed by the addition of substrate and incubation for 1 hour. Enzyme activity was determined by fluorescence measurement of AMC and expressed as percentage enzyme activity relative to the control without inhibitor. (A) Bestatin inhibition of HEK293T cell extract assayed with Ala-AMC. (B) Bestatin inhibition of HEK293T cell extract assayed with Leu-AMC. (C) Puromycin inhibition of HEK293T cell extract assayed with Ala-AMC. (D) Puromycin inhibition of HEK293T cell extract assayed with Leu-AMC. (E) Puromycin inhibition of purified PSAP assayed with Ala-AMC. (F) Puromycin inhibition of purified PSAP assayed with Leu-AMC. Error bars show standard error of mean (n = 4), points without error bars had error ranges smaller than the symbol size. Statistical analysis was performed using Student’s t-test: *, p≤0.05; **, p≤0.01; ***, p≤0.001; ns, no significant difference (p>0.05) versus the control.

Aminopeptidases that remove single amino acids from peptides are thought to play major roles in intracellular peptide degradation; these enzymes include LAP, PSAP, and bleomycin hydrolase, all of which cleave a variety of amino acids including both Ala and Leu (which are commonly found on the N-terminus of the peptides detected in the peptidomics analysis). To determine if any of these aminopeptidases are present in HEK293T cells, the cell extracts were incubated with either Ala-AMC or Leu-AMC in the absence and presence of various inhibitors. Both bestatin and puromycin inhibited >80% of the cleavage of either substrate (Figure 7). This suggests that PSAP is the major aminopeptidase capable of cleaving Ala-AMC and Leu-AMC in HEK293T cell extracts; LAP is inhibited by bestatin but not puromycin, while bleomycin hydrolase is not inhibited by either compound. The potency of puromycin as an inhibitor of the HEK293T cell extract is comparable to its potency as an inhibitor of purified PSAP (Figure 7).

**Figure 8:**
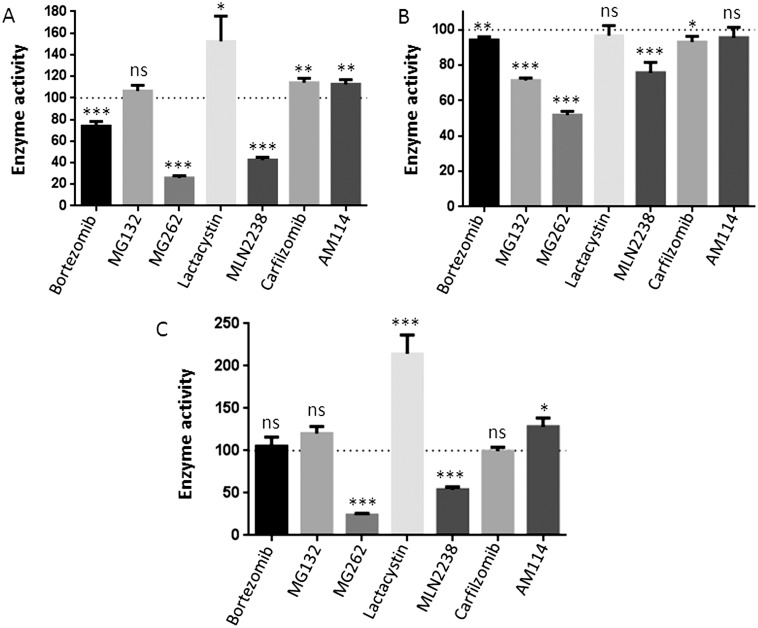
Effect of proteasome inhibitors on Ala-AMC and Leu-AMC cleavage. HEK 293T cell extract or recombinant PSAP was treated with 10 µM of each of the proteasome inhibitors for 1 hour in addition to a 30 min pre-incubation without the substrate. Enzyme activity was determined by fluorescence measurement of AMC and expressed as percentage enzyme activity relative to the control without inhibitor. Panels A and B represent the effect of proteasome inhibitors on cleavage of Ala-AMC and Leu-AMC respectively, in HEK293T cell extract. Panel C shows effect of the inhibitors on cleavage of Ala-AMC by purified PSAP. Error bars show standard error of mean (n = 4). Statistical analysis was performed using Student’s t-test: *, p≤0.05; **, p≤0.01; ***, p≤0.001; ns, no significant difference (p>0.05) versus the control.

Cleavage of Ala-AMC and Leu-AMC by the HEK293T cell extracts is partially inhibited by 10 µM bortezomib (Figure 8A, B). Two of the other boronate-containing compounds (MG262 and MLN2238) also inhibit the cleavage of these two substrates, but the di-boronate compound AM114 is without effect (Figure 8A, B). This suggests that the effect is not simply due to the presence of a boronate group. Other proteasome inhibitors tested in this study either showed no effect or a slight increase or decrease, but these changes were not consistent with the two different substrates (Figure 8). The proteasome inhibitors were also tested with purified PSAP; while MG262 and MLN2238 were inhibitory, bortezomib had no significant effect (Figure 8C). Because the inhibition seen with 10 µM bortezomib was 25%, and this was close to the residual amount of activity in cells treated with 50 µM bestatin or puromycin (∼20%), one possible explanation was that bortezomib was a strong inhibitor of other cellular aminopeptidases that contributed to cleavage of Ala-AMC and which were not inhibited by high concentrations of bestatin or puromycin. To test this, HEK293T cell extracts were assayed with Ala-AMC in the absence or presence of high concentrations of bestatin, and with 10 or 50 µM bortezomib. There was no statistical difference between the activity measured in the presence of 500 µM bestatin alone and the activity measured with 50 µM bestatin together with either 10 or 50 µM bortezomib (Table 4). Thus, bortezomib does not appear to inhibit the bestatin-insensitive aminopeptidase activity of HEK293T cells. The effects of bortezomib on cellular aminopeptidase activity are likely to be secondary effects on the PSAP, and not due to inhibition of another cellular aminopeptidase detected with the Ala-AMC or Leu-AMC substrates.

**Table 4:** Enzyme activity of HEK293T cell extracts assayed with Ala-AMC in the absence or presence of various inhibitors.

Bestatin	Bortezomib	Enzyme activity (% control with no inhibitor)
500 µM	0	15.5±0.7
50 µM	10 µM	14.8±0.6
50 µM	50 µM	14.8±0.8

**Figure 9:**
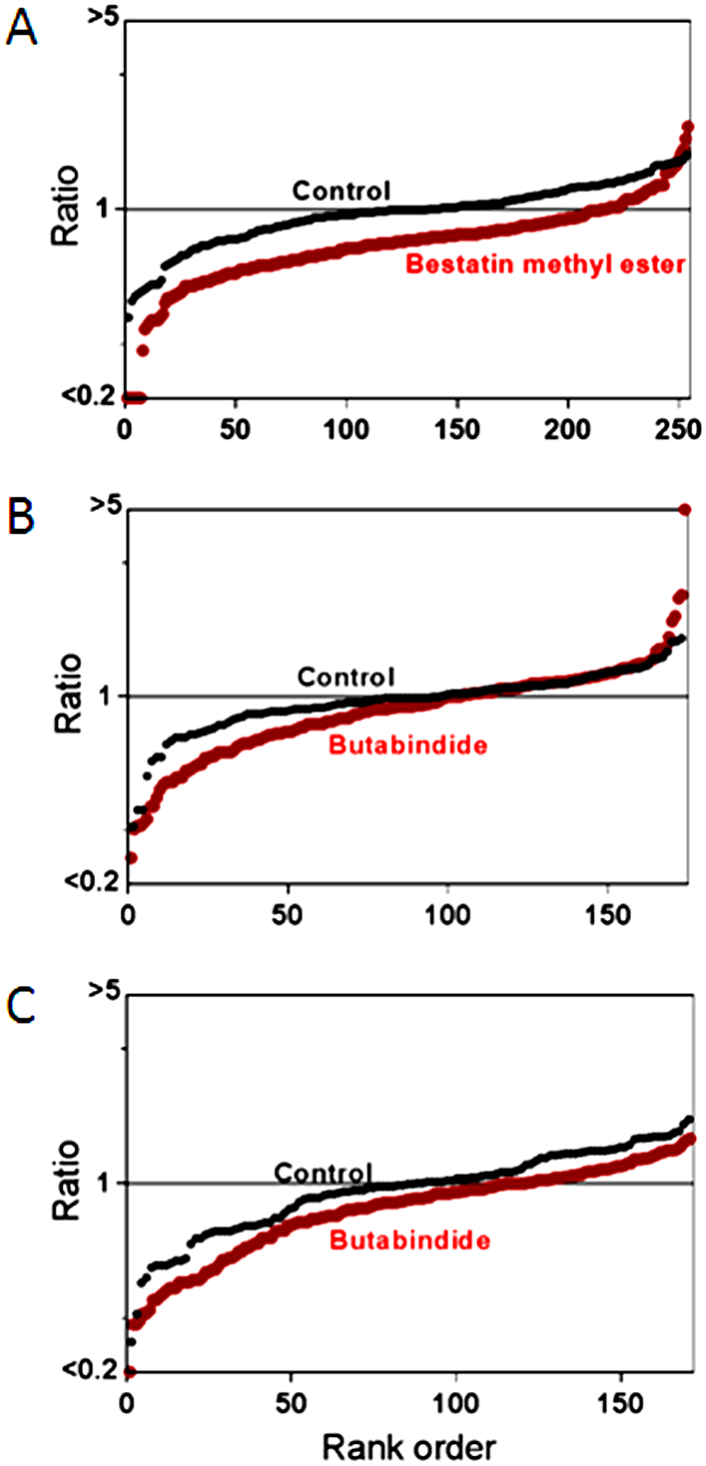
Summary plots of the peptidome of HEK293T cells in response to aminopeptidase inhibitors. Panels A–C represent data from experiments on HEK293T cells treated with (A) 100 µM bestatin methyl ester for 75 min; (B) 100 µM butabindide for 35 min; (C) 100 µM butabindide for 75 min. Specifications and criteria for the summary plots are described in Figure 3 legend.

**Figure 10:**
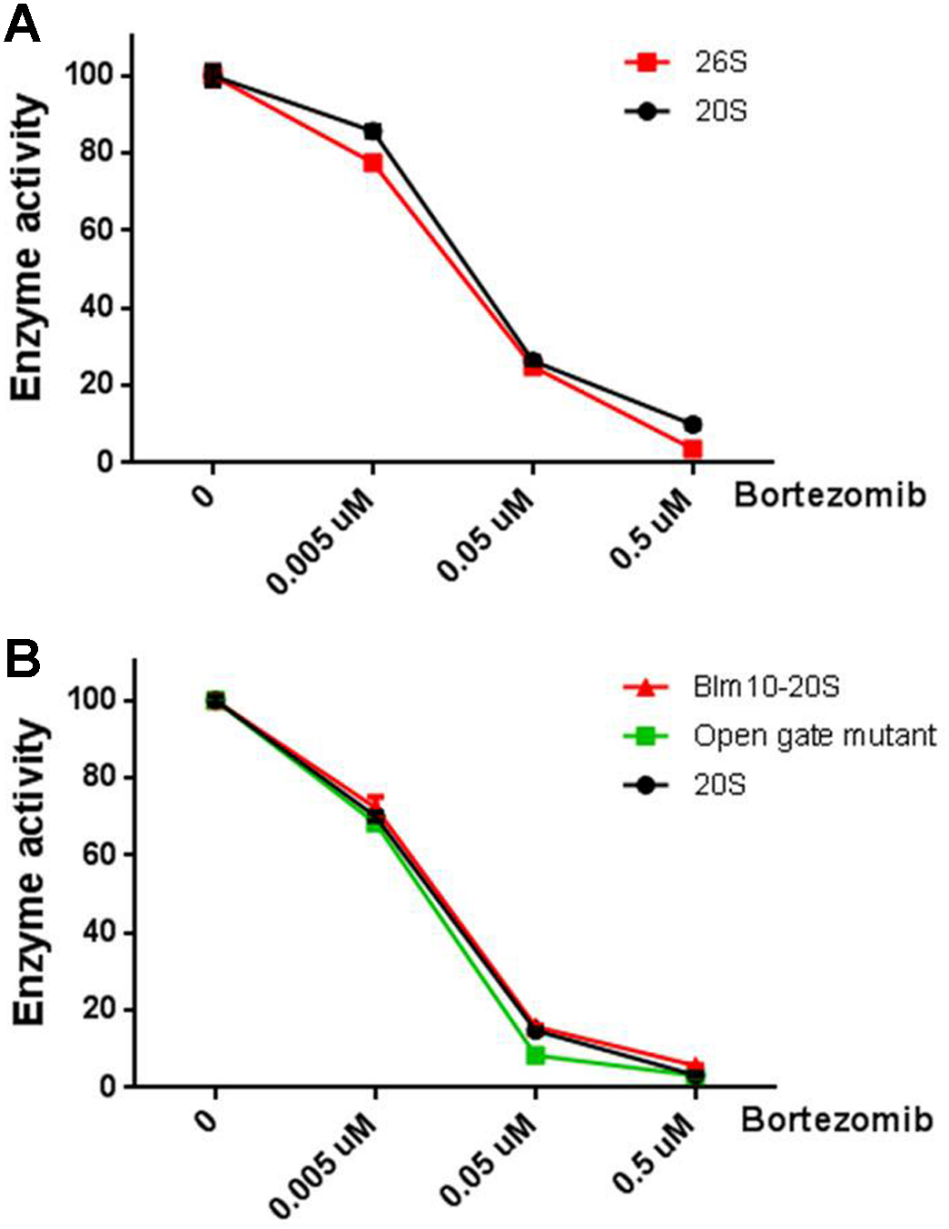
Effect of bortezomib on the chymotryptic-like activity of different forms of the proteasome from yeast. Purified 20S subunit, Blm10-20S, open gate mutant of the 20S subunit and 26S were combined with the indicated concentrations of bortezomib in the presence of substrate. After incubation for 15 minutes, enzyme activity was measured by monitoring the fluorescence kinetics for the next 30 minutes. Panel A represents inhibition of 26S and 20S by bortezomib in the presence of buffer containing ATP. Panel B represents inhibition of Blm10-20S, the open gate mutant and 20S by bortezomib in the presence of buffer without ATP. Enzyme activity is expressed as percent control without inhibitor. Error bars show standard error of the mean; error bars smaller than symbol size are not visible.

To directly test whether PSAP or LAP contribute to the degradation of the observed intracellular peptides, we performed peptidomic analysis after treatment of HEK293T cells with bestatin or bestatin methyl ester, a variant that has a higher cell permeability than bestatin. Neither bestatin (Table S1) nor bestatin methyl ester (Figure 9A and Table S1) dramatically alter the cellular peptidome. Similarly, butabindide treatment of HEK293T cells also failed to substantially alter the peptide levels (Figure 9B, C and Table S1), consistent with a previous report that TPP2 is not involved in the production of peptides that bind to MHC class I proteins [41]. The results of these studies suggest that neither PSAP nor LAP contribute to the degradation of the intracellular peptides detected in the peptidomics analyses. We therefore considered the possibility that the observed peptides are degraded by certain forms of the proteasome such as the 20S core particle alone, or the 20S core particle complex with PA200/Blm 10. This latter form is able to degrade peptides and small proteins with unstructured regions but not ubiquitinated proteins [34], [42]. If the peptides we observe in our studies are degraded by the 20S core particle (alone or in complex with PA200/Blm 10), and if this activity is more sensitive to bortezomib than the 26S proteasome, then this could account for the bortezomib-induced increase in peptides levels. To test this, we compared the effect of bortezomib on the chymotryptic-like activity of various proteasome forms purified from yeast. The 26S proteasome and the 20S proteasome core particle were compared in the presence of ATP; both showed comparable inhibition by bortezomib (Figure 10A). In the absence of ATP, the 20S core particle was compared to the Blm10-activated 20S core particle and to an open gate mutant of the 20S core particle; bortezomib inhibited all three forms with comparable potency (Figure 10B). Therefore, it does not appear that bortezomib has a differential effect on the various forms of the proteasome.

## Discussion

Proteins are converted into peptides by the proteasome, and it was generally assumed that the resulting peptides were rapidly degraded into amino acids by cellular peptidases [6], [11]. Peptidomic analyses of mouse brain, originally developed to detect neuropeptides, also found numerous peptides that were derived from cytosolic, nuclear, and mitochondrial proteins, collectively termed “intracellular” peptides [12], [13], [16]. The majority of the intracellular peptides detected in mouse brain and human cell lines are not derived from the most abundant proteins or from the most unstable proteins, suggesting that these peptides are not merely protein degradation fragments awaiting further degradation by aminopeptidases [13]. Instead, it was proposed that these peptides were either selectively generated (independent of protein degradation) or were selectively retained, possibly through binding to intracellular proteins [13]. It has been proposed that some of these intracellular peptides are able to modulate protein-protein interactions or perform other physiological functions [12], [16]. An important question has been the enzymatic pathways responsible for the formation and degradation of the intracellular peptides.

Two previous studies tested the effect of various proteasome inhibitors on the cellular peptidome [18], [19]. In one study, epoxomicin was found to reduce the levels of most peptides, consistent with a role for the proteasome in the production of these peptides [19]. The other study tested bortezomib and found that a large number of peptides were elevated by this proteasome inhibitor [18]. A major finding of the present study is that two other proteasome inhibitors, MG262 and carfilzomib, produce an increase in many of the intracellular peptides previously found to be elevated by bortezomib. Both bortezomib and MG262 are boronate-containing compounds but carfilzomib is not. Furthermore, two other boronate-containing compounds (MLN2238 and AM114) did not produce the same effect as MG262 and bortezomib. Instead, MLN2238 produced changes in peptide levels that were more like those caused by non-boronate compounds such as epoxomicin, clasto-Lactacystin β-lactone, and MG132. AM114 did not substantially alter the cellular peptidome at the concentration tested, consistent with the inability of this compound to inhibit the proteasome at low micromolar concentrations.

The present results, together with the previous findings, present a paradox–how can proteasome inhibitors cause an increase in the levels of many intracellular peptides? If these peptides are produced by the proteasome, it would be expected that proteasome inhibitors would cause a decrease in their levels. Although bortezomib and MG262 led to an increase in the largest number of peptides, all of the effective proteasome inhibitors tested in the present study produced unexpected increases in the levels of some peptides. The most likely explanation of this apparent paradox is that bortezomib and other proteasome inhibitors have allosteric effects that alter the specificity or the stability of the proteasome. Increasing evidence suggests that some proteasome inhibitors exibit an allosteric effect on proteasome stability; MG262 treated purified 26S proteasomes were resistant to apyrase-induced proteasome dissociation whereas MG132 had no effect on proteasome stability [22]. In other studies, bortezomib was reported to activate the beta 2 subunit, which cleaves at basic amino acids [23]–[25]. The previous peptidomic study with epoxomicin noted that many of the peptides which were elevated by this compound contained an acidic residue in the P1 position of the cleavage site required to produce these peptides [19]. Because epoxomicin does not inhibit the beta 1 subunit responsible for cleavage at acidic residues, it would be expected that inhibition of the beta 2 and beta 5 subunits would lead to a greater share of protein degradation occurring at acidic residues. However, some of the peptides that were elevated upon treatment of cells with epoxomicin, and most of the peptides elevated upon treatment of cells with bortezomib, have hydrophobic residues in the P1 position of the cleavage site [18], [19]. Similarly, carfilzomib and MG262 also elevated levels of peptides that required cleavage at hydrophobic sites; all of these inhibitors are most potent at the beta 5 subunit, which is responsible for cleaving at hydrophobic sites. Somehow the inhibitors of the beta 5 subunit appear to be activating the beta 5 subunit, possibly by affecting the opening of the gate within the 20S proteasome core particle; bortezomib, MG262, and epoxomicin were all found to open this gate [26]. In the present study, we found that bortezomib showed comparable inhibition of the 20S core particle and an open-gate mutant of this 20S core particle when assayed with the standard substrate for beta 5 activity (Succ-Leu-Leu-Val-Tyr-AMC), but it is possible that allosteric regulation of the proteasome affects the intracellular peptides differently than the synthetic substrate. For example, Kisselev et al found that hydrophobic peptides including Succ-Leu-Leu-Val-Tyr-AMC can trigger gate opening and stimulate the activity of 20S particles [43]. A related possibility is that the various proteasome forms are differentially affected by inhibitors. In support of this hypothesis, the antiviral drug ritonavir was found to activate the chymotryptic-like activity of the 26S form of the proteasome while inhibiting the 20S form [44]. Although we found no difference in the effect of bortezomib on the chymotryptic-like activity of the 26S versus the 20S form, or the 20S form activated by Blm10, it remains possible that allosteric effects of the proteasome inhibitors influence cleavage of the intracellular peptides by the various proteasome forms.

Our results do not support the hypothesis that the proteasome inhibitors have off-target effects on enzymes that further degrade the peptides produced by the proteasome (Figure 1). A number of different enzymes have been implicated in the degradation of proteasome products, including oligopeptidases, aminopeptidases, and TPP2 [4]–[7]. Based on a bioinformatic approach, it was proposed that bortezomib could be an inhibitor of TPP2, although no direct evidence of this was provided [21]. In the present study, we could not detect any inhibition of TPP2 activity by bortezomib. Furthermore, peptidomic analysis of cells treated with butabindide, a potent and selective TPP2 inhibitor, did not produce dramatic changes in the cellular peptidome. These results suggest that the bortezomib-induced changes in the cellular peptidome are not due to inhibition of TPP2. The failure of butabindide to cause large changes in the cellular peptidome suggests that TPP2 does not play a major role in the degradation of the intracellular peptides detected with the peptidomic technique, consistent with the finding that TPP2 is not required for the production of peptides bound to HLA [41]. Our observation that bortezomib is a weak inhibitor of aminopeptidase activity in HEK293T cells was initially considered to be consistent with this off-target explanation. However, there are many problems with this interpretation. First, bortezomib did not inhibit purified PSAP, which was the major Ala-AMC and Leu-AMC-cleaving aminopeptidase detected in HEK293T cell extracts. Second, although MG262 and MLN2238 also inhibited the HEK293T cell aminopeptidase activity and purified PSAP, only MG262 caused a large increase in many of the intracellular peptides; MLN2238 did not show this effect. Finally, neither bestatin nor bestatin methyl ester caused a large change in the levels of intracellular peptides; bestatin inhibits PSAP as well as other aminopeptidases. The absence of large changes in peptide levels in response to treatment with these inhibitors suggests that neither PSAP nor other bestatin-sensitive enzymes contribute to the degradation of the intracellular peptides observed in this study. This finding is consistent with the observation that mice lacking either LAP or PSAP show normal processing and presentation of peptides in complex with MHC class I molecules [45], [46].

Previous studies investigating peptides bound to MHC class I molecules tested the origin of these peptides by treating cells with proteasome inhibitors and measuring levels of HLA-bound peptides. One study found 104 different peptides bound to HLA-B27, and although the majority was decreased by treatment of cells with epoxomicin, 31 peptides were not affected more than 20% and were therefore considered to be proteasome independent [47]. A subsequent study examining peptides bound to other HLA proteins also found a substantial number of peptides that were not affected by treatment with either epoxomicin or MG132 [48]. Many of these proteasome-independent peptides arose from small basic proteins (size <16.5 kDa and isoelectric point >7). In the present study, only three peptides were consistently found to be resistant to the various proteasome inhibitors (Table 2; note that although there are four peptides listed in this table, thymosin beta-10 is a small protein, not a proteasome product). The three proteins that give rise to the peptides in Table 2 range in size from 63 to 272 amino acids. This is comparable to the size range of the proteins listed in Table 1 (83–286 amino acids) and Table 3 (69–294 amino acids). Furthermore, basic proteins are not more common than acidic proteins in Tables 2 and 3. Therefore, the tendency for proteasome-independent HLA-bound peptides to be products of basic proteins is not shared by the proteasome-independent peptides found in whole cell extracts in the present study. On the other hand, all of the proteins listed in Tables 1–3 are under 300 amino acids in length, which is well below the size of the average protein encoded by the human genome [47].

Milner and colleagues examined the effect of epoxomicin and bortezomib on the rate of synthesis of HLA-bound peptides and cellular proteins in MCF-7 cells [49]. Although the rate of synthesis of many HLA-bound peptides was decreased when cells were treated with the proteasome inhibitors for 4 hours, other peptides showed no effect or even an increase in their rates of synthesis in response to the proteasome inhibitors [49]. Similarly, the rate of cellular protein synthesis was generally decreased for most proteins, but some were not affected or had elevated rates of synthesis. A comparison of the proteins listed in the supplemental data Table S2A of Milner et al [49] with the proteins found in the present study revealed ten proteins in common for which data were available for both epoxomicin and bortezomib. Two of these proteins showed a decrease in levels of intracellular peptides in our analysis and also a decrease in protein synthesis (gene names PARK7 and RBM3). Another protein (gene name PEBP1) showed a decrease in intracellular peptides and protein synthesis with epoxomicin and no substantial change with bortezomib (within 10% of the DMSO control). However, none of the other seven proteins showed a correlation between the rate of protein synthesis and the levels of intracellular peptides after treatment with bortezomib or epoxomicin; gene names of these proteins are PPIA, TMSB10, EIF5A, ERH, MIF, UBA52, and RPLP2. Therefore, the changes in protein synthesis observed by Milner et al cannot account for the altered levels of intracellular peptides observed in the present study.

The therapeutic effect of bortezomib and carfilzomib as anticancer drugs is generally considered to be through alteration of protein turnover. However, these drugs produce a rapid and dramatic change in the cellular peptidome, increasing the levels of some peptides and decreasing the levels of other peptides. If these peptides are biologically active, the changes in peptide levels could contribute to the physiological effects of the drugs. Several studies have shown that intracellular peptides can influence signal transduction pathways [14], [15], [50]. Many other studies have shown that synthetic peptides of 10–20 amino acids can perturb a number of processes within the cell [51], [52]. Therefore, it is possible that the therapeutic and/or side effects of bortezomib and carfilzomib are mediated in part through the changes in the cellular peptidome.

## Supporting Information

Table S1 **Summary of peptidomic results from experiments involving proteasome inhibitors and other treatments of HEK293T and SH-SY5Y cells.** This table lists each peptide observed in peptidomic studies, including both identified and unknown peptides. It contains >6000 rows, with each row representing a peptide in a particular experimental run.
(XLSX)

Table S2 **Data for heatmap figure.** This table lists the peptides used for Figure 5, including peptide sequences, cleavage sites, and relative levels in treated versus control replicates.
(XLSX)

## References

[1] Hershko A, Ciechanover A (1992) The ubiquitin system for protein degradation. Annu.Rev.Biochem. 61: 761–807. 10.1146/annurev.bi.61.070192.003553 1323239

[2] Goldberg AL (2003) Protein degradation and protection against misfolded or damaged proteins. Nature 426: 895–9. 14685250 10.1038/nature02263

[3] Nussbaum AK, Dick TP, Keilholz W, Schirle M, Stevanovic S, et al (1998) Cleavage motifs of the yeast 20S proteasome beta subunits deduced from digests of enolase 1. Proc.Natl.Acad.Sci. USA 95: 12504–9. 10.1073/pnas.95.21.12504 PMC22860 9770515

[4] Saric T, Graef CI, Goldberg AL (2004) Pathway for degradation of peptides generated by proteasomes: a key role for thimet oligopeptidase and other metallopeptidases. J. Biol.Chem. 279: 46723–32. 10.1074/jbc.M406537200 15328361

[5] Chang SC, Momburg F, Bhutani N, Goldberg AL (2005) The ER aminopeptidase, ERAP1, trims precursors to lengths of MHC class I peptides by a “molecular ruler” mechanism. Proc.Natl.Acad.Sci. U.S.A 102: 17107–12. 10.1073/pnas.0500721102 PMC1287962 16286653

[6] Reits E, Neijssen J, Herberts C, Benckhuijsen W, Janssen L, et al (2004) A major role for TPPII in trimming proteasomal degradation products for MHC class I antigen presentation. Immunity. 20: 495–506. 10.1016/s1074-7613(04)00074-3 15084277

[7] Kessler BM, Glas R, Ploegh HL (2002) MHC class I antigen processing regulated by cytosolic proteolysis-short cuts that alter peptide generation. Mol.Immunol. 39: 171–9. 10.1016/s0161-5890(02)00100-1 12200049

[8] Nolte WM, Tagore DM, Lane WS, Saghatelian A (2009) Peptidomics of Prolyl Endopeptidase in the Central Nervous System. Biochem. 48: 11971–81. 10.1021/bi901637c PMC2813186 19911840

[9] Tagore DM, Nolte WM, Neveu JM, Rangel R, Guzman-Rojas L, et al (2009) Peptidase substrates via global peptide profiling. Nature Chem.Biol. 5: 23–5. 10.1038/nchembio.126 PMC2730040 19011639

[10] Goldberg AL, Cascio P, Saric T, Rock KL (2002) The importance of the proteasome and subsequent proteolytic steps in the generation of antigenic peptides. Mol.Immunol. 39: 147–64. 10.1016/s0161-5890(02)00098-6 12200047

[11] Reits E, Griekspoor A, Neijssen J, Groothuis T, Jalink K, et al (2003) Peptide diffusion, protection, and degradation in nuclear and cytoplasmic compartments before antigen presentation by MHC class I. Immunity. 18: 97–108. 10.1016/s1074-7613(02)00511-3 12530979

[12] Fricker LD (2010) Analysis of mouse brain peptides using mass spectrometry-based peptidomics: implications for novel functions ranging from non-classical neuropeptides to microproteins. Mol.Biosyst. 6: 1355–65. 10.1039/c003317k PMC2902593 20428524

[13] Gelman JS, Sironi J, Castro LM, Ferro ES, Fricker LD (2011) Peptidomic analysis of human cell lines. J. Proteome.Res. 10: 1583–92. 10.1021/pr100952f PMC3070057 21204522

[14] Russo LC, Asega AF, Castro LM, Negraes PD, Cruz L, et al (2012) Natural intracellular peptides can modulate the interactions of mouse brain proteins and thimet oligopeptidase with 14-3-3 epsilon and calmodulin. Proteomics 12: 2641–55. 22740335 10.1002/pmic.201200032

[15] Cunha FM, Berti DA, Ferreira ZS, Klitzke CF, Markus RP, et al (2008) Intracellular peptides as natural regulators of cell signaling. J. Biol.Chem. 283: 24448–59. 10.1074/jbc.M801252200 PMC3259820 18617518

[16] Ferro ES, Hyslop S, Camargo AC (2004) Intracellullar peptides as putative natural regulators of protein interactions. J. Neurochem. 91: 769–77. 10.1111/j.1471-4159.2004.02757.x 15525330

[17] de Araujo CB, Russo LC, Castro LM, Forti FL, do Monte ER, et al (2014) A novel intracellular peptide derived from G1/S cyclin D2 induces cell death. J. Biol.Chem. 10.1074/jbc.M113.537118 PMC4059116 24764300

[18] Gelman JS, Sironi J, Berezniuk I, Dasgupta S, Castro LM, et al (2013) Alterations of the intracellular peptidome in response to the proteasome inhibitor bortezomib. PloS One 8: e53263. 23308178 10.1371/journal.pone.0053263 PMC3538785

[19] Fricker LD, Gelman JS, Castro LM, Gozzo FC, Ferro ES (2012) Peptidomic analysis of HEK293T cells: effect of the proteasome inhibitor epoxomicin on intracellular peptides. J. Proteome Res. 11: 1981–90. 10.1021/pr2012076 PMC3315381 22304392

[20] Adams J, Kauffman M (2004) Development of the proteasome inhibitor Velcade (Bortezomib). Cancer Invest. 22: 304–11. 10.1081/cnv-120030218 15199612

[21] Arastu-Kapur S, Anderl JL, Kraus M, Parlati F, Shenk KD, et al (2011) Nonproteasomal targets of the proteasome inhibitors bortezomib and carfilzomib: a link to clinical adverse events. Clinical Cancer Res. 17: 2734–43. 10.1158/1078-0432.CCR-10-1950 21364033

[22] Kleijnen MF, Roelofs J, Park S, Hathaway NA, Glickman M, et al (2007) Stability of the proteasome can be regulated allosterically through engagement of its proteolytic active sites. Nature Struct. Molec. Biol. 14: 1180–8. 10.1038/nsmb1335 18026118

[23] Crawford LJ, Walker B, Ovaa H, Chauhan D, Anderson KC, et al (2006) Comparative selectivity and specificity of the proteasome inhibitors BzLLLCOCHO, PS-341, and MG-132. Cancer Res. 66: 6379–86. 10.1158/0008-5472.CAN-06-0605 16778216

[24] Moravec RA, O’Brien MA, Daily WJ, Scurria MA, Bernad L, et al (2009) Cell-based bioluminescent assays for all three proteasome activities in a homogeneous format. Anal.Biochem. 387: 294–302. 10.1016/j.ab.2009.01.016 19454251

[25] Lightcap ES, McCormack TA, Pien CS, Chau V, Adams J, et al (2000) Proteasome inhibition measurements: clinical application. Clin.Chem. 46: 673–83. 10794750

[26] Osmulski PA, Hochstrasser M, Gaczynska M (2009) A tetrahedral transition state at the active sites of the 20S proteasome is coupled to opening of the alpha-ring channel. Structure 17: 1137–47. 19679091 10.1016/j.str.2009.06.011 PMC2746003

[27] Beck P, Dubiella C, Groll M (2012) Covalent and non-covalent reversible proteasome inhibition. Biol. Chem. 393: 1101–20. 10.1515/hsz-2012-0212 23091276

[28] Kuhn DJ, Chen Q, Voorhees PM, Strader JS, Shenk KD, et al (2007) Potent activity of carfilzomib, a novel, irreversible inhibitor of the ubiquitin-proteasome pathway, against preclinical models of multiple myeloma. Blood 110: 3281–90. 17591945 10.1182/blood-2007-01-065888 PMC2200918

[29] Ostrowska H, Wojcik C, Omura S, Worowski K (1997) Lactacystin, a specific inhibitor of the proteasome, inhibits human platelet lysosomal cathepsin A-like enzyme. Biochem. Biophys. Res. Comm. 234: 729–32. 10.1006/bbrc.1997.6434 9175783

[30] Morano C, Zhang X, Fricker LD (2008) Multiple Isotopic Labels for Quantitative Mass Spectrometry. Anal.Chem. 80: 9298–309. 10.1021/ac801654h PMC2771887 19551992

[31] Berti DA, Morano C, Russo LC, Castro LM, Cunha FM, et al (2009) Analysis of intracellular substrates and products of thimet oligopeptidase (EC 3.4.24.15) in human embryonic kidney 293 cells. J. Biol.Chem. 284: 14105–16. 10.1074/jbc.M807916200 PMC2682859 19282285

[32] Che FY, Zhang X, Berezniuk I, Callaway M, Lim J, et al (2007) Optimization of neuropeptide extraction from the mouse hypothalamus. J. Proteome.Res. 6: 4667–76. 10.1021/pr060690r 17979226

[33] Zhang X, Che FY, Berezniuk I, Sonmez K, Toll L, et al (2008) Peptidomics of Cpe(fat/fat) mouse brain regions: implications for neuropeptide processing. J.Neurochem. 107: 1596–613. 10.1111/j.1471-4159.2008.05722.x PMC2663970 19014391

[34] Dange T, Smith D, Noy T, Rommel PC, Jurzitza L, et al (2011) Blm10 protein promotes proteasomal substrate turnover by an active gating mechanism. J. Biol.Chem. 286: 42830–9. 10.1074/jbc.M111.300178 PMC3234834 22025621

[35] Leggett DS, Hanna J, Borodovsky A, Crosas B, Schmidt M, et al (2002) Multiple associated proteins regulate proteasome structure and function. Mol.Cell 10: 495–507. 12408819 10.1016/s1097-2765(02)00638-x

[36] Berkers CR, Verdoes M, Lichtman E, Fiebiger E, Kessler BM, et al (2005) Activity probe for in vivo profiling of the specificity of proteasome inhibitor bortezomib. Nat.Methods 2: 357–62. 15846363 10.1038/nmeth759

[37] Chauhan D, Tian Z, Zhou B, Kuhn D, Orlowski R, et al (2011) In vitro and in vivo selective antitumor activity of a novel orally bioavailable proteasome inhibitor MLN9708 against multiple myeloma cells. Clin. Cancer Res. 17: 5311–21. 10.1158/1078-0432.CCR-11-0476 PMC3156932 21724551

[38] Gelman JS, Wardman JH, Bhat VB, Gozzo FC, Fricker LD (2012) Quantitative peptidomics to measure neuropeptide levels in animal models relevant to psychiatric disorders. Methods Mol.Biol. 829: 487–503. 10.1007/978-1-61779-458-2_31 PMC4327865 22231834

[39] Lyons PJ, Fricker LD (2011) Peptidomic approaches to study proteolytic activity. Current Protocols Prot. Sci. Chapter 18: Unit18.13. 10.1002/0471140864.ps1813s65 PMC3184789 21842468

[40] Geier E, Pfeifer G, Wilm M, Lucchiari-Hartz M, Baumeister W, et al (1999) A giant protease with potential to substitute for some functions of the proteasome. Science 283: 978–81. 9974389 10.1126/science.283.5404.978

[41] Marcilla M, Villasevil EM, de Castro JA (2008) Tripeptidyl peptidase II is dispensable for the generation of both proteasome-dependent and proteasome-independent ligands of HLA-B27 and other class I molecules. Eur. J. Immunol. 38: 631–9. 10.1002/eji.200737444 18286573

[42] Qian MX, Pang Y, Liu CH, Haratake K, Du BY, et al (2013) Acetylation-mediated proteasomal degradation of core histones during DNA repair and spermatogenesis. Cell 153: 1012–24. 23706739 10.1016/j.cell.2013.04.032 PMC3983474

[43] Kisselev AF, Kaganovich D, Goldberg AL (2002) Binding of hydrophobic peptides to several non-catalytic sites promotes peptide hydrolysis by all active sites of 20 S proteasomes - Evidence for peptide-induced channel opening in the alpha-rings. J. Biol.Chem. 277: 22260–70. 10.1074/jbc.M112360200 11927581

[44] Gaedicke S, Firat-Geier E, Constantiniu O, Lucchiari-Hartz M, Freudenberg M, et al (2002) Antitumor effect of the human immunodeficiency virus protease inhibitor ritonavir: induction of tumor-cell apoptosis associated with perturbation of proteasomal proteolysis. Cancer Res. 62: 6901–8. 12460905

[45] Towne CF, York IA, Neijssen J, Karow ML, Murphy AJ, et al (2008) Puromycin-sensitive aminopeptidase limits MHC class I presentation in dendritic cells but does not affect CD8 T cell responses during viral infections. J. Immunol. 180: 1704–12. 10.4049/jimmunol.180.3.1704 18209067

[46] Towne CF, York IA, Neijssen J, Karow ML, Murphy AJ, et al (2005) Leucine aminopeptidase is not essential for trimming peptides in the cytosol or generating epitopes for MHC class I antigen presentation. J. Immunol. 175: 6605–14. 10.4049/jimmunol.175.10.6605 16272315

[47] Marcilla M, Cragnolini JJ, de Castro JAL (2007) Proteasome-independent HLA-B27 Ligands arise mainly from small basic proteins. Mol.Cell.Proteomics 6: 923–38. 17308301 10.1074/mcp.M600302-MCP200

[48] Garcia-Medel N, Sanz-Bravo A, Barnea E, Admon A, de Castro JAL (2012) The Origin of Proteasome-inhibitor Resistant HLA Class I Peptidomes: a Study With HLA-A*68:01. Mol.Cell.Proteomics M111.011486. 10.1074/mcp.M111.011486 PMC3270105 21969608

[49] Milner E, Gutter-Kapon L, Bassani-Strenberg M, Barnea E, Beer I, et al (2013) The effect of proteasome inhibition on the generation of the human leukocyte antigen (HLA) peptidome. Mol.Cell.Proteomics 12: 1853–64. 23538226 10.1074/mcp.M112.026013 PMC3708171

[50] Berti DA, Russo LC, Castro LM, Cruz L, Gozzo FC, et al (2012) Identification of intracellular peptides in rat adipose tissue: insights into insulin resistance. Proteomics 12: 2668–81. 22740317 10.1002/pmic.201200051

[51] Rubinstein M, Niv MY (2009) Peptidic modulators of protein-protein interactions: progress and challenges in computational design. Biopolymers 91: 505–13. 19226619 10.1002/bip.21164

[52] Arkin MR, Whitty A (2009) The road less traveled: modulating signal transduction enzymes by inhibiting their protein-protein interactions. Curr.Opin.Chem.Biol. 13: 284–90. 10.1016/j.cbpa.2009.05.125 19553156

